# Biomarkers of Chondrocyte Apoptosis and Autophagy in Osteoarthritis

**DOI:** 10.3390/ijms160920560

**Published:** 2015-08-31

**Authors:** Giuseppe Musumeci, Paola Castrogiovanni, Francesca Maria Trovato, Annelie Martina Weinberg, Mohammad K. Al-Wasiyah, Mohammed H. Alqahtani, Ali Mobasheri

**Affiliations:** 1 Department of Biomedical and Biotechnological Sciences, Human Anatomy and Histology Section, School of Medicine, University of Catania, Catania 95123, Italy; E-Mail: pacastro@unict.it; 2 Department of Clinical and Experimental Medicine, Internal Medicine Division, School of Medicine, University of Catania, Catania 95123, Italy; E-Mail: trovatofrancesca@gmail.com; 3 Department of Orthopaedic Surgery, Medical University of Graz, 8036 Graz, Austria; E-Mail: annelie.weinberg@t-online.de; 4 Aziziah Maternity and Children’s Hospital, Jeddah 50204, Saudi Arabia; E-Mails: alwasiyah@doctor.com (M.K.A.-W.); mhalqahtani@kau.edu.sa (M.H.A.); 5 King Fahd Medical Research Center (KFMRC), King AbdulAziz University, Jeddah 21589, Saudi Arabia; 6 The D-BOARD European Consortium for Biomarker Discovery, Department of Veterinary Preclinical Sciences, School of Veterinary Medicine, Faculty of Health and Medical Sciences, University of Surrey, Guildford GU2 7XH, UK; E-Mail: a.mobasheri@surrey.ac.uk; 7 Arthritis Research UK Centre for Sport, Exercise and Osteoarthritis, Arthritis Research UK Pain Centre, Medical Research Council and Arthritis Research UK Centre for Musculoskeletal Ageing Research, University of Nottingham, Queen’s Medical Centre, Nottingham NG7 2UH, UK

**Keywords:** osteoarthritis (OA), chondrocyte death, apoptosis, autophagy, biomarker

## Abstract

Cell death with morphological and molecular features of apoptosis has been detected in osteoarthritic (OA) cartilage, which suggests a key role for chondrocyte death/survival in the pathogenesis of OA. Identification of biomarkers of chondrocyte apoptosis may facilitate the development of novel therapies that may eliminate the cause or, at least, slow down the degenerative processes in OA. The aim of this review was to explore the molecular markers and signals that induce chondrocyte apoptosis in OA. A literature search was conducted in PubMed, Scopus, Web of Science and Google Scholar using the keywords chondrocyte death, apoptosis, osteoarthritis, autophagy and biomarker. Several molecules considered to be markers of chondrocyte apoptosis will be discussed in this brief review. Molecular markers and signalling pathways associated with chondroycte apoptosis may turn out to be therapeutic targets in OA and approaches aimed at neutralizing apoptosis-inducing molecules may at least delay the progression of cartilage degeneration in OA.

## 1. Introduction

Articular cartilage is an avascular tissue whose functional properties of mechanical support and joint lubrication are dependent on the functional integrity of its extracellular matrix (ECM). Cartilage ECM is rich in fibrillar collagens, especially type II, large aggregating proteoglycans and smaller hydrophilic macromolecules that confer lubricative and swelling properties to the tissue [1]. Under normal physiological conditions chondrocytes maintain a delicate equilibrium between the synthesis and degradation of the ECM components, thus regulating the structural and functional integrity of cartilage [2]. Cartilage is an avascular tissue with limited regenerative ability. Furthermore, in degenerative joint diseases there is evidence of cell death in chondrocytes, with consequent repercussions on tissue maintenance and functionality. Apoptotic cell death has been detected in osteoarthritic (OA) cartilage; this was associated with matrix degradation and calcification, which suggests a role for cell death/survival in OA pathogenesis. In contrast to necrotic cell death, apoptosis is programmed, orderly and does not induce inflammation. Apoptosis requires energy and is generally important for tissue homeostasis during the life course of an individual [3]. Apoptosis represents a form of programmed cell death in which a pre-determined sequence of events leads to the elimination of old, unnecessary and unhealthy cells, without releasing harmful substances into the surrounding area [4]. Cells that are undergoing apoptosis exhibit a characteristic pattern of morphologic changes, including cell shrinkage, condensation, fragmentation of the nucleus and bubbling of the plasma membrane, known as “blebbing”, chromatin condensation and nucleosomal fragmentation [5]. The crucial function of apoptotic mechanisms in cartilage degeneration and the involvement of apoptosis in various conditions associated with OA have been thoroughly explored [6,7,8,9].

## 2. Methods

In this review, we analysed published articles from the most recent literature, providing a balanced and comprehensive overview of the most important discoveries in relation to pathogenesis and possible biomarkers and target molecules for the treatment of OA. Subsequently the selected articles were divided in “Osteoarthritis”, “Chondrocytes apoptosis in Osteoarthritis”, “Biomarkers of chondrocyte degeneration” and “Autophagy”, to structure the review and render it more understandable for interested researchers by providing a detailed and schematic overview of the most relevant studies that have been done in this field. The literature search and the manuscript writing were done between December 2014 and August 2015. The databases used were PubMed, Scopus, Web of Science and Google Scholar using appropriate keywords (chondrocyte death, apoptosis, osteoarthritis, autophagy, biomarkers). Out of approximately 220 papers (original articles, systematic and meta-analysis reviews), 81 were chosen and considered appropriate for this focused review. Other papers, related to the chosen keywords, were discarded, as they were considered to be outside the scope of the research. The time period chosen for the literature search was from 1998 until 2015. The bibliographic research has been divided into four different steps and has followed an inductive reasoning. In the first step, the research was focused on papers regarding “osteoarthritis”; in the second step the “Chondrocytes apoptosis in Osteoarthritis”; in the third step the “Biomarkers of chondrocyte degeneration” and in the fourth step the “Autophagy”. The overall research was focused on both *in vitro* and *in vivo* studies, and on the analysis of the obtained data.

## 3. Chondrocyte Apoptosis in Osteoarthritis

**Figure 1:**
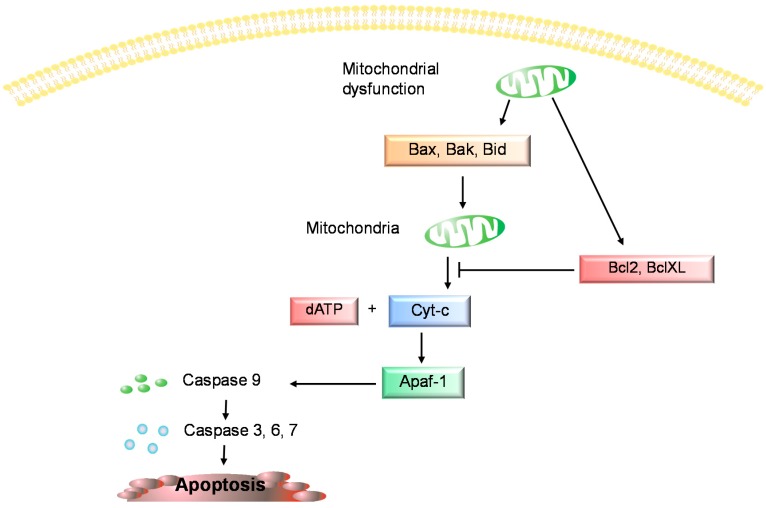
The intrinsic pathway of apoptosis. Bcl-2: B-cell lymphoma-2; Bcl-XL: B-cell lymphoma-XL; Bax: Bcl-2-associated X protein; Bak: Bcl-2 homologous antagonist/killer; Bid: BH3 interacting domain death agonist; dATP: deoxyadenosine triphosphate; Apaf-1: apoptotic protease activating factor.

Apoptosis has been positively correlated with the severity of cartilage destruction and matrix depletion in human osteoarthritic tissue specimens [1]. Freshly isolated chondrocytes from human OA cartilage exhibited morphological evidence of apoptosis, clear cytoplasmic, cell-surface blebs, altered nuclear shape, apoptotic bodies and a parallel loss of nuclear volume. Chondrocytes from normal donors did not show any cytoplasmic features of apoptotic cell death. These findings suggest that the OA chondrocytes demonstrate differences in predisposition towards apoptosis [4,7]. In general studies of cartilage apoptosis several molecules are considered to be potential biomarkers but in the current literature only a few of them are discussed in this context. The studies published thus far have used a wide range of techniques, such as histology, terminal deoxynucleotidyl trasferase dUTP nick end labelling (TUNEL), analysis of caspase-3 expression, enzyme linked immunosorbent assays (ELISA), anti-poly (ADPribose) polymerase (anti-PARP), p85 and fluorescence activated cell sorter analysis (FACS) to examine the relationship between apoptosis and chondrocytes death in OA. In several studies, electron microscopy was used to identify ultrastructural changes attributable to apoptosis in chondrocytes within osteoarthritic cartilage [6]. Apoptosis is induced through two main, alternative pathways: death receptor-mediated (or extrinsic) and mitochondria-dependent (or intrinsic), both lead to the activation of executor caspases [4,5,6,7]. Caspases are a group of intracellular cysteine protease enzymes that destroy essential cellular proteins, leading to controlled cell death. There are two types of caspase enzymes: initiator caspases (caspases 2, 8, 9, and 10), activated through the apoptosis-signaling pathways, that activate the effector caspases (caspases 3, 6, and 7), which, in an expanding cascade, carry out apoptosis [10,11,12]. Caspase 3 promotes the typical apoptosis features, including DNA fragmentation and cell death in many tissues including cartilage [9,10,11,12]. The intrinsic pathway of apoptosis is regulated by mitochondrial parameters [13,14,15]. Mitochondrial mediated apoptosis may initiate through the release of pro-apoptotic proteins into the cytosol due to mitochondrial dysfunction [13,14,15]. However, mitochondria also contain anti-apoptotic proteins [14]. Mitochondrial pro- and anti-apoptotic proteins belong to the B-cell lymphoma-2 (Bcl-2) family, and the balance between them controls apoptosis [14,16]. The anti-apoptotic proteins Bcl-2 and Bcl-XL inhibit cytochrome c (cyt-c) release, whereas Bcl-2-associated X protein (Bax), Bcl-2 homologous antagonist/killer (Bak), and BH3 interacting domain death agonist (Bid), all pro-apoptotic proteins, promote its release from mitochondria. Cyt-c and deoxyadenosine triphosphate (dATP) bind to apoptotic protease activating factor (Apaf-1) to form a multimeric complex that recruits and activates procaspase 9, that in turn activates caspase 3, resulting in cell apoptosis (Figure 1) [3,17]. The extrinsic pathway of apoptosis is activated by pro-apoptotic receptors on the cell surface [18].

## 4. Biomarkers of Chondrocyte Degeneration

**Figure 2:**
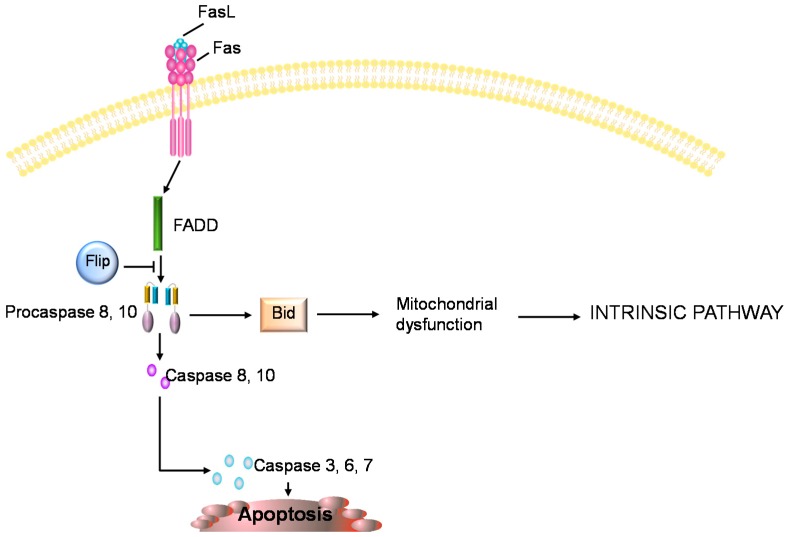
The extrinsic pathway of apoptosis. FasL: death-inducing molecule Fas ligand; FADD: Fas-associated death domain; Flip: FLICE inhibitory protein; Bid: BH3 interacting domain death agonist.

The molecular signals involved in the cartilage degeneration that is typical of OA are numerous and act via specific pathways that are shared with other molecules. Current OA biomarker research has focused on investigating the molecular markers involved in the degeneration of cartilage in OA, in order to develop new diagnostic and prognostic assays and facilitate the development of novel disease modifying therapies. In this brief review we report on molecular biomarkers of chondrocyte apoptosis in order to expand the knowledge that is currently available in this topic and provide a fresh perspective for apoptosis and biomarker researchers. Several studies have demonstrated that nitric oxide (NO) plays a role in chondrocyte apoptosis [19,20]. NO is thought to operate through a mitochondria-dependent mechanism, enhancing the expression of pro-inflammatory cytokines involved in the breakdown of cartilage ECM [21,22,23,24]. Chemical NO donors such as sodium nitroprusside (SNP) induce cell death in cultured human chondrocytes [24]. Incubation of human articular chondrocytes with SNP has been shown to increase caspase-3 and caspase-7 gene expression and downregulate Bcl-2 mRNA levels, which is characteristic of apoptosis [21,22,23,24]. SNP can induce apoptosis of human chondrocytes through cytoskeletal remodeling, mitogen-activated protein kinase kinase kinase-1/c-Jun N-terminal kinase (MEKK1/JNK) activation, Bax translocation, mitochondrial dysfunction and sequential caspase activation (caspase-9, -3 and -6), leading to DNA fragmentation [20,21]. NO may be blocked by reactive oxygen species (ROS), and the balance between intracellular NO and ROS seems to determine if chondrocytes die through apoptosis or necrosis; indeed when the concentration of ROS is low, apoptosis occurs in the presence of NO. In contrast, high concentrations of ROS promote necrosis [25]. Incubation with NO alone does not induce apoptotic cell death in chondrocytes [25,26]. However, many reports indicate that NO, as a catabolic factor in OA, can induce cell death [25,26]. Another study showed that apoptotic bodies isolated from NO-treated chondrocytes contain alkaline phosphatase, nucleoside triphosphate (NTP) pyrophosphohydrolase activities and can precipitate calcium [27,28], and it is well known that calcium signalling is involved in almost cellular functions including metabolism, proliferation, differentiation, and also apoptosis [20,29]. NO was shown to cause cell death and induce the tumour suppressor protein p53 via p38 mitogen-activated protein kinase (MAPK) and nuclear factor κB (NF-κB) [21,30]. In addition to p53, c-myc may also regulate chondrocyte death in OA. In lesions of arthritic cartilage, *in situ* nick-end labeling (ISNEL), which identifies apoptotic cells, is associated with the expression of p53 and c-myc [31], and in a canine model of OA, high levels of c-myc were shown in cartilage erosions [32]. Another important biomarker of chondrocyte apoptosis is the death receptor Fas that induces cell death following ligation with Fas ligand (FasL) [33]. Fas-mediated chondrocyte loss may contribute to cartilage degradation in OA and rheumatoid arthritis (RA) [20,27,28]. Fas ligation by FasL is followed by recruitment of Fas-associated death domain (FADD) and subsequently of caspase 8 activation, which can be inhibited by the anti-apoptotic molecule FLICE inhibitory protein (Flip). Caspase 8 induces apoptosis by directly activating caspase 3 or by cleaving Bid, resulting in mitochondrial dysfunction and subsequent release of cyt-c and activation of caspases 9 and 3 (Figure 2) [3,6]. Poly (ADP-ribose) polymerase 1 (PARP-1) is a nuclear DNA repair enzyme that is implicated in DNA repair and maintenance of genomic integrity. In apoptosis, PARP-1 is cleaved and inactivated by caspase-3, resulting in the formation of an N-terminal fragment containing most of the DNA binding domain and a C-terminal fragment containing the catalytic domain [10,11,12]. Thus, the presence of C-terminal (89 kDa) PARP-1 fragment is considered an important biomarker of apoptosis [10,11,12,34]. Authors investigated the histological aspects of the growth plate after injury and the relative caspase-3 expression by immunohistochemistry and histomorphometry; moreover western blot analysis was carried out to quantify the expression of caspase-3 and cleaved PARP-1. They concluded that caspase-3 and cleaved PARP-1 expression significantly increases in growth plate chondrocytes with a time-course progression [9,10,35]. A recent study showed that Toll-like receptors 1/2 (TLR1/2) were expressed in degenerated chondrocytes in OA, and may react to cartilage matrix/chondrocyte-derived danger signals or degradation products. This leads to synthesis of pro-inflammatory cytokines, which stimulate further TLRs and cytokine expression, establishing a vicious circle. They also demonstrated that tumour necrosis factor-α (TNF-α) treatment increased TLR1 and TLR2 mRNA expression [36,37,38]. If TNF-α mediates chondrocyte death is under observation. Data in literature showed that TNF-α stimulation of chondrocytes led to a small increase in the number of TUNEL- or ISNEL-positive cells [21,39]. Moreover, DNA fragmentation in response to TNF-α was detected with a sensitive ELISA based technique when the chondrocytes were simultaneously stimulated with proteasome inhibitors [40]. Therefore, TNF-α alone may have no effect on apoptosis [21]. However, TNF-α in combination with actinomycin-D or Ro 31–8220 induces an increase in caspase-1 and -8 mRNA and protein levels [41,42]. Instead, the transforming growth factor beta (TGF-β) in articular cartilage can work via two pathways, the ALK5/Smad2/3P and the ALK1/Smad1/5/8P route, the first being protective and the latter favouring chondrocyte terminal differentiation [43]. Normally TGF-β has chondroprotective capabilities, but under specific conditions it should determine OA like changes in healthy articular cartilage [44,45], shifting the molecular pathway from ALK5/Smad2/3P to ALK1/Smad1/5/8P, favouring terminal differentiation of chondrocytes and apoptosis [43,46]. Various inflammatory conditions, including arthritis, are characterized by cartilage degradation through ECM destruction that should be also mediated by the small calcium-binding S100 proteins [21,47]. The interaction of small calcium-bindingA4 (S100A4) with receptor for advanced glycation end products (RAGE) increases matrix metalloproteinase (MMP)-13 production in cartilage [47], and upregulates MMP-13 and other MMPs in RA derived synovial fibroblasts [48]. S100A4 was reported to bind tumour suppressor protein p53 and to regulate its function [49], possibly promoting apoptosis. In OA cartilage histological analysis has revealed structural alterations and histochemical results have confirmed the presence of matrix calcification and reduction in proteoglycans, which reflects the presence of pathological changes. Moreover, these data coincided with an immunohistochemical increase in apoptotic cells, when compared to normal cartilage [4,7]. The inflammation process causes stress to chondrocytes and induces cell death as a biological defense mechanism; on the other hand, survival of new chondrocytes increases in order to maintain cell homeostasis [4,7,50,51]. Our hypothesis is that chondrocyte apoptosis could be secondary to cartilage degradation. It is supported by the fact that cell-matrix interaction is vital for chondrocyte survivability. Chondrocyte survival is thought to be mediated by integrins connecting the ECM components, like collagen, laminin and fibronectin, to various intracellular cytoskeletal proteins [6]. Loss of this adhesion may trigger chondrocyte apoptosis. It is likely that disruption in chondrocyte-matrix interaction is either due to direct injury to the cartilage, causing biochemical changes or loss of ECM (e.g., denaturation of type II collagen, changes in fibronectin and other matrix protein expressions) [6]. Moreover, the extent of chondrocyte apoptosis is positively correlated with expression of fibronectin, one of the key ECM molecules involved in communication between the cartilage cells and surrounding matrix, and up-regulation of expression of which is associated with the severity of articular cartilage damage [8]. Decreased expression or availability of important ECM macromolecules in cartilage is sufficient to induce chondrocyte apoptosis and cause exacerbation of matrix damage [6,8]. It has been observed that TNF-α-stimulation can cause dose-dependent depletion of proteoglycans [36]. A chondroprotective agent is the mucinous glycoprotein product of the proteoglycan 4 (PRG4) gene, called lubricin [52,53]. Lubricin is responsible for the boundary lubrication of articular cartilage [52,53] and other joint parts [54,55,56,57]. Thanks to its boundary-lubricating properties, lubricin prevents synoviocyte overgrowth, protects cartilage surfaces and prevents cartilage wear [58]. Drug therapy with glucocorticoids (GCs) decreases the expression of lubricin and increases the expression of caspase-3 in rats, determining increased friction in the joint. Following physical activity the values return to normal levels compared to controls [50,59]. Mechanical stimulation is able to stimulate release of lubricin in articular cartilage and to inhibit caspase-3 activity, preventing chondrocyte death [50,60]. Nevertheless mechanical injury has been demonstrated to induce cell death and cartilage matrix degradation in bovine and human cartilage, through the release of ROS and proteoglycans, and the production of inflammatory and catabolic factors, such as MMPs, NO, “a disintegrin and metalloprotease with thrombospondin type I repeats-5” (ADAMTS-5) and interleukin-1β (IL-1β), [21,61,62,63,64]. Moreover, western blot analysis revealed that static load-induced chondrocyte apoptosis was accompanied by increased phosphorylation of JNK, extracellular signal-regulated kinase 1/2 (ERK1/2), and p38 mitogen-activated protein kinase (MAPK). So the mitochondrial pathway is involved in mechanical stress-induced chondrocyte apoptosis [65]. Glucocorticoids activate the caspase cascade and trigger Bax-mediated mitochondrial apoptosis in growth plate chondrocytes, causing growth retardation in young mice, indeed dexamethasone was found to increase the pro-apoptotic proteins Bcl-xS, Bad, and Bak as well as the proteolysis of Bid [50,60,66]. Syndecan-4 is a transmembrane heparan sulfate proteoglycan and its immunostaining is abundant in both human and murine OA cartilage [46,67]. *In vitro* studies have identified direct interactions between syndecan-4 and ADAMTS-5, and it is claimed that ADAMTS-5 activity, causing aggrecan breakdown in OA [68], is dependent on MMP-3, mediating collagen type II breakdown and cartilage erosion, and the latter activity is controlled by syndecan-4 [67,69,70].

## 5. Autophagy

**Figure 3:**
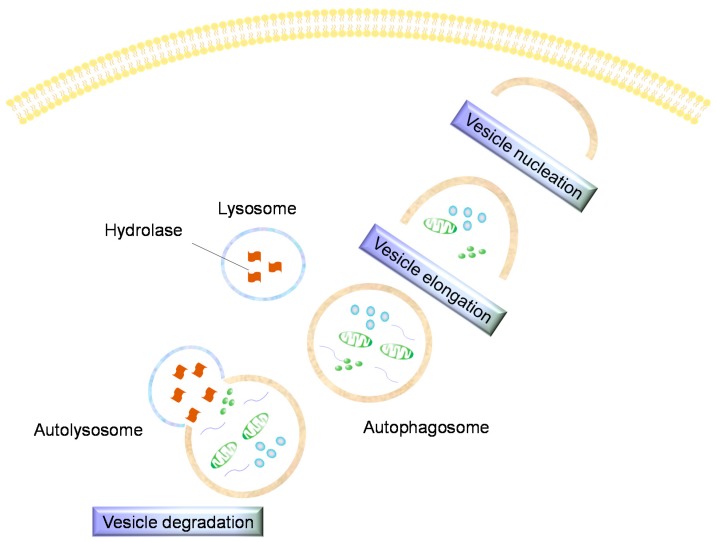
Key features of autophagy. Cytoplasmic and intracellular organelles are sequestered within autophagic vacuoles to be degraded by lysosomes.

Autophagy is a self-degradative process that is important for balancing sources of energy at critical times during development and in response to cell stress. Although autophagy is not a type of cell death, it is important for cartilage homeostasis. Generally autophagy promotes cell survival by adapting cells to stress conditions, but this process has also been considered as a non-apoptotic cell death program [71,72,73]. Although autophagy shares some characteristics with apoptosis, such as absence of inflammation during the death process as well as ATP consumption, apoptosis is an essential physiological process that plays a critical role in development and tissue homeostasis, whereas autophagy means literally, to eat oneself, and in most circumstances, autophagy promotes cell survival by adapting cells to stress conditions, but at the same time, this process has also been considered as a non-apoptotic cell death program [72]. During autophagy, parts of the cytoplasm and intracellular organelles are sequestered within characteristic autophagic vacuoles and are subsequently degraded by lysosomes. Recent data support the idea that autophagy can occur in combination with apoptosis in OA [21,72], indeed Almonte-Becerril and collaborators demonstrated that in early stages of OA, chondrocytes from the superficial zone showed an increased expression of both apoptotic cell death and autophagic markers, even if authors suggested that autophagy is activated as an adaptive response to sublethal conditions, with the aim to avoid cell death. Afterward, as the degenerative process progresses, the conjunctly expression of both death markers in chondrocytes from both the superficial and middle zones, can be related with a defect in the reparation response of chondrocytes, due the increased activation of death signals, as well as the catabolic mechanisms prevalence. On the other hand, in the deep zone, authors evidenced the absence of autophagy and the increased apoptosis, that can be associated with the substitution of chondrocytes linked to the abnormal calcification of the cartilage present in late stage of OA. Therefore, authors concluded that the functional relationship between apoptosis and autophagy during OA pathogenesis is complex in the sense that, in early stages of OA, autophagy probably could be activated as an adaptive response that avoids cell death, whereas in late stages of OA, this process also could be conjunctly activated with apoptosis as an alternative pathway to cellular demise [72]. Further studies are requires to precise and confirm the role of autophagic cell death, in the different stages of cartilage breakdown during the experimental OA model [21,72]. In induced murine OA, it was shown that decreased expression of the autophagy markers correlate with proteoglycan loss and an increase in the levels of the apoptosis marker PARP p85 [46,74]. In addition, in OA, autophagy is associated with an increased chondrocyte apoptosis and a reduction and loss of Unc-51-like kinase 1 (ULK1), an inducer of autophagy, Beclin1, a regulator of autophagy, and microtubule associated protein 1 light chain 3 (LC3), which executes autophagy (Figure 3, Table 1) [20,74]. Autophagy can be inhibited by interleukins cleaved by Nod-like receptor protein 3 (NLRP3)-dependent caspase-1 [20,75,76]. Moreover several autophagy-inducing agents are object of recent researches as protective for OA. Among them, Tougu Xiaotong capsule inhibits tidemark replication and cartilage degradation by regulating chondrocyte autophagy and it could be a potential therapeutic agent for the reduction of cartilage degradation that occurs in osteoarthritis [77]. Manipulation of Hypoxia-inducible factor 1-alpha (HIF-1α) and 2-alpha (HIF-2α) has been suggested as a promising approach to the treatment of OA. Indeed the increased HIF-1α and HIF-2α mediate the response of chondrocytes to hypoxia. HIF-1α regulates both autophagy and apoptosis, promoting the chondrocyte phenotype, maintaining chondrocyte viability, and supporting metabolic adaptation to a hypoxic environment. In contrast HIF-2α induces the expression of catabolic factors in chondrocytes, and enhances Fas expression leading to chondrocyte apoptosis and regulates autophagy in maturing chondrocytes [78]. Recent studies showed that rapamycin activates autophagy in human chondrocytes preventing the development of OA *in vitro*, while the systemic and/or intra-articular injection of rapamycin reduces the severity of experimental osteoarthritis *in vivo* [79], representing a potential therapeutic approach to prevent OA. Also glucosamine modulates and enhances autophagy pathway *in vitro* and *in vivo*, warranting other studies on the efficacy of glucosamine in OA [80].

**Table 1:** Key features of different types of cell death.

Apoptosis	Necrosis	Autophagy
Rounding of cells; Plasma membrane blebbing; Nuclear fragmentation; Chromatin condensation; Reduction in cellular and nuclear volume; Apoptosis body formation; Mitochondrial swelling (rare)	Plasma membrane rupture; Mitochondrial and cytoplasmic swelling; No vesicle formation; Moderate chromatin condensation	Accumulation of autophagic vacuoles; Lack of chromatin condensation; Late-stage mitochondrial swelling

## 6. Conclusions

The diversity of the molecular signals that can induce cell death in chondrocytes in OA lead us to hypothesize that future therapeutic approaches should be aimed at neutralizing untimely and excessive apoptosis in articular cartilage. Developing novel biological and pharmaceutical agents that can counteract apoptosis and neutralise apoptosis-inducing molecules could potentially delay the progression of cartilage degeneration in OA. Identification of target molecules for gene therapy or biological or chemical reagent delivery to target sites could help prevent cartilage degeneration. Further longer-term *in vitro*, *in vivo* and clinical studies are needed to understand the precise role of apoptosis in cartilage degeneration mechanisms in OA and other osteoarticular disorders.

## References

[1] Musumeci G., Aiello F.C., Szychlinska M.A., di Rosa M., Castrogiovanni P., Mobasheri A. (2015). Osteoarthritis in the XXIst century: Risk factors and behaviours that influence disease onset and progression. Int. J. Mol. Sci..

[2] Di Rosa M., Szychlinska M.A., Tibullo D., Malaguarnera L., Musumeci G. (2014). Expression of CHI3L1 and CHIT1 in osteoarthritic rat cartilage model. A morphological study. Eur. J. Histochem..

[3] Galanti C., Musumeci G., Valentino J., Giunta S., Castorina S. (2013). A role for apoptosis in temporomandibular joint disc degeneration. A contemporary review. Ital. J. Anat. Embryol..

[4] Musumeci G., Loreto C., Carnazza M.L., Martinez G. (2011). Characterization of apoptosis in articular cartilage derived from the knee joints of patients with osteoarthritis. Knee Surg. Sports Traumatol. Arthrosc..

[5] Huppertz B., Frank H.G., Kaufmann P. (1999). The apoptosis cascade—Morphological and immunohistochemical methods for its visualization. Anat. Embryol..

[6] Zamli Z., Sharif M. (2011). Chondrocyte apoptosis: A cause or consequence of osteoarthritis?. Int. J. Rheum. Dis..

[7] Musumeci G., Loreto C., Carnazza M.L., Strehin I., Elisseeff J. (2011). OA cartilage derived chondrocytes encapsulated in poly (ethylene glycol) diacrylate (PEGDA) for the evaluation of cartilage restoration and apoptosis in an *in vitro* model. Histol. Histopathol..

[8] Thomas C.M., Murray R., Sharif M. (2011). Chondrocyte apoptosis determined by caspase-3 expression varies with fibronectin distribution in equine articular cartilage. Int. J. Rheum. Dis..

[9] Musumeci G., Castrogiovanni P., Mazzone V., Szychlinska M.A., Castorina S., Loreto C. (2014). Histochemistry as a unique approach for investigating normal and osteoarthritic cartilage. Eur. J. Histochem..

[10] Musumeci G., Castrogiovanni P., Loreto C., Castorina S., Pichler K., Weinberg A.M. (2013). Post-traumatic caspase-3 expression in the adjacent areas of growth plate injury site. A morphological study. Int. J. Mol. Sci..

[11] Shakibaei M., John T., Seifarth C., Mobasheri A. (2007). Resveratrol inhibits IL-1β-induced stimulation of caspase-3 and cleavage of PARP in human articular chondrocytes *in vitro*. Ann. N. Y. Acad. Sci..

[12] Giunta S., Castorina A., Marzagalli R., Szychlinska M.A., Pichler K., Mobasheri A., Musumeci G. (2015). Ameliorative effects of PACAP against cartilage degeneration. Morphological, immunohistochemical and biochemical evidence from *in vivo* and *in vitro* models of rat osteoarthritis. Int. J. Mol. Sci..

[13] Fulda S., Debatin K.M. (2004). Apoptosis signaling in tumor therapy. Ann. N. Y. Acad. Sci..

[14] Ganguly R., Pierce G.N. (2015). The toxicity of dietary trans fats. Food Chem. Toxicol..

[15] Chabi B., Ljubicic V., Menzies K.J., Huang J.H., Sallem A., Hood D.A. (2008). Mitochondrial function and apoptotic susceptibility in aging skeletal muscle. Aging Cell.

[16] Akl H., Vervloessem T., Kiviluoto S., Bittremieux M., Parys J.B., De Smedt H., Bultynck G. (2014). A dual role for the anti-apoptotic Bcl-2 protein in cancer: Mitochondria *versus* endoplasmic reticulum. Biochim. Biophys. Acta.

[17] Loreto C., Rapisarda V., Carnazza M.L., Musumeci G., D’Agata V., Valentino M., Martinez G. (2007). Bitumen products alter Bax, Bcl-2 and cytokeratin expression: An *in vivo* study of chronically exposed road pavers. J. Cutan. Pathol..

[18] Ghobrial I.M., Witzig T.E., Adjei A.A. (2005). Targeting apoptosis pathways in cancer therapy. CA Cancer J. Clin..

[19] Takahashi K., Hashimoto S., Kubo T., Hirasawa Y., Lotz M., Amiel D. (2000). Effect of hyaluronan on chondrocyte apoptosis and nitric oxide production in experimentally induced osteoarthritis. J. Rheumatol..

[20] Mobasheri A., Matta C., Zákány R., Musumeci G. (2015). Chondrosenescence: Definition, hallmarks and potential role in the pathogenesis of osteoarthritis. Maturitas.

[21] Grogan S.P., D’Lima D.D. (2010). Joint aging and chondrocyte cell death. Int. J. Clin. Rheumtol..

[22] Wu G.J., Chen T.G., Chang H.C., Chiu W.T., Chang C.C., Chen R.M. (2007). Nitric oxide from both exogenous and endogenous sources activates mitochondria-dependent events and induces insults to human chondrocytes. J. Cell. Biochem..

[23] Maneiro E., Lopez-Armada M.J., de Andres M.C., Caramés B., Martín M.A., Bonilla A., del Hoyo P., Galdo F., Arenas J., Blanco F.J. (2005). Effect of nitric oxide on mitochondrial respiratory activity of human articular chondrocytes. Ann. Rheum. Dis..

[24] Cherng Y.G., Chang H.C., Lin Y.L., Kuo M.L., Chiu W.T., Chen R.M. (2008). Apoptotic insults to human chondrocytes induced by sodium nitroprusside are involved in sequential events, including cytoskeletal remodeling, phosphorylation of mitogen-activated protein kinase kinase kinase-1/c-Jun N-terminal kinase, and Bax-mitochondria-mediated caspase activation. J. Orthop. Res..

[25] Kuhn K., Hashimoto S., Lotz M. (2000). IL-1β protects human chondrocytes from CD95-induced apoptosis. J. Immunol..

[26] Del Carlo M., Loeser R.F. (2002). Nitric oxide-mediated chondrocyte cell death requires the generation of additional reactive oxygen species. Arthritis Rheum..

[27] Hashimoto H., Tanaka M., Suda T., Tomita T., Hayashida K., Takeuchi E., Kaneko M., Takano H., Nagata S., Ochi T. (1998). Soluble Fas ligand in the joints of patients with rheumatoid arthritis and osteoarthritis. Arthritis Rheum..

[28] Hashimoto S., Ochs R.L., Rosen F., Quach J., McCabe G., Solan J., Seegmiller J.E., Terkeltaub R., Lotz M. (1998). Chondrocyte-derived apoptotic bodies and calcification of articular cartilage. Proc. Natl. Acad. Sci. USA.

[29] Berridge M.J., Lipp P., Bootman M.D. (2000). The versatility and universality of calcium signalling. Nat. Rev. Mol. Cell Biol..

[30] Kim S.J., Hwang S.G., Shin D.Y., Kang S.S., Chun J.S. (2002). P38 kinase regulates nitric oxide-induced apoptosis of articular chondrocytes by accumulating p53 via NFkappa B-dependent transcription and stabilization by serine 15 phosphorylation. J. Biol. Chem..

[31] Yatsugi N., Tsukazaki T., Osaki M., Koji T., Yamashita S., Shindo H. (2000). Apoptosis of articular chondrocytes in rheumatoid arthritis and osteoarthritis: Correlation of apoptosis with degree of cartilage destruction and expression of apoptosis-related proteins of p53 and c-myc. J. Orthop. Sci..

[32] Pelletier J.P., Faure M.P., DiBattista J.A., Wilhelm S., Visco D., Martel-Pelletier J. (1993). Coordinate synthesis of stromelysin, interleukin-1, and oncogene proteins in experimental osteoarthritis. An immunohistochemical study. Am. J. Pathol..

[33] Loreto C., Barbagli G., Djinovic R., Vespasiani G., Carnazza M.L., Miano R., Musumeci G., Sansalone S. (2011). Tumor necrosis factor-related apoptosis-inducing ligand (TRAIL) and its death receptor (DR5) in Peyroniet’s disease. A Biomolecular Study of Apoptosis Activation. J. Sex. Med..

[34] Shakibaei M., Csaki C., Nebrich S., Mobasheri A. (2008). Resveratrol suppresses interleukin-1β-induced inflammatory signaling and apoptosis in human articular chondrocytes: Potential for use as a novel nutraceutical for the treatment of osteoarthritis. Biochem. Pharmacol..

[35] Mobasheri A., Kalamegam G., Musumeci G., Batt M.E. (2014). Chondrocyte and mesenchymal stem cell-based therapies for cartilage repair in osteoarthritis and related orthopaedic conditions. Maturitas.

[36] Sillat T., Barreto G., Clarijs P., Soininen A., Ainola M., Pajarinen J., Korhonen M., Konttinen Y.T., Sakalyte R., Hukkanen M. (2013). Toll-like receptors in human chondrocytes and osteoarthritic cartilage. Acta Orthop..

[37] Bobacz K., Sunk I.G., Hofstaetter J.G., Amoyo L., Toma C.D., Akira S., Weichhart T., Saemann M., Smolen J.S. (2007). Toll-like receptors and chondrocytes: The lipopolysaccharide-induced decrease in cartilage matrix synthesis is dependent on the presence of toll-like receptor 4 and antagonized by bone morphogenetic protein 7. Arthritis Rheum..

[38] Liu-Bryan R. (2013). Synovium and the innate inflammatory network in osteoarthritis progression. Curr. Rheumatol. Rep..

[39] Fischer B.A., Mundle S., Cole A.A. (2000). Tumor necrosis factor-α induced DNA cleavage in human articular chondrocytes may involve multiple endonucleolytic activities during apoptosis. Microsc. Res. Tech..

[40] Kühn K., Lotz M. (2001). Regulation of CD95 (Fas/APO-1)-induced apoptosis in human chondrocytes. Arthritis Rheum..

[41] López-Armada M.J., Caramés B., Lires-Deán M., Cillero-Pastor B., Ruiz-Romero C., Galdo F., Blanco F.J. (2006). Cytokines, tumor necrosis factor-α and interleukin-1β, differentially regulate apoptosis in osteoarthritis cultured human chondrocytes. Osteoarthr. Cartil..

[42] Caramés B., López-Armada M.J., Cillero-Pastor B., Lires-Dean M., Vaamonde C., Galdo F., Blanco F.J. (2008). Differential effects of tumor necrosis factor-α and interleukin-1β on cell death in human articular chondrocytes. Osteoarthr. Cartil..

[43] Madej W., van Caam A., Blaney Davidson E.N., van der Kraan P.M., Buma P. (2014). Physiological and excessive mechanical compression of articular cartilage activates Smad2/3P signaling. Osteoarthr. Cartil..

[44] Itayem R., Mengarelli-Widholm S., Reinholt F.P. (1999). The long-term effect of a short course of transforming growth factor-β1 on rat articular cartilage. APMIS.

[45] Van Beuningen H.M., Glansbeek H.L., van der Kraan P.M., van den Berg W.B. (2000). Osteoarthritis-like changes in the murine knee joint resulting from intra-articular transforming growth factor-β injections. Osteoarthr. Cartil..

[46] Van den Berg W.B. (2011). Osteoarthritis year 2010 in review: Pathomechanisms. Osteoarthr. Cartil..

[47] Yammani R.R., Long D., Loeser R.F. (2009). Interleukin-7 stimulates secretion of S100A4 by activating the JAK/STAT signaling pathway in human articular chondrocytes. Arthritis Rheum..

[48] Senolt L., Grigorian M., Lukanidin E., Simmen B., Michel B.A., Pavelka K., Gay R.E., Gay S., Neidhart M. (2006). S100A4 is expressed at site of invasion in rheumatoid arthritis synovium and modulates production of matrix metalloproteinases. Ann. Rheum. Dis..

[49] Grigorian M., Andresen S., Tulchinsky E., Kriajevska M., Carlberg C., Kruse C., Cohn M., Ambartsumian N., Christensen A., Selivanova G. (2001). Tumor suppressor p53 protein is a new target for the metastasis-associated Mts1/S100A4 protein: Functional consequences of their interaction. J. Biol. Chem..

[50] Musumeci G., Loreto C., Leonardi R., Castorina S., Giunta S., Carnazza M.L., Trovato F.M., Pichler K., Weinberg A.M. (2013). The effects of physical activity on apoptosis and lubricin expression in articular cartilage in rats with glucocorticoid-induced osteoporosis. J. Bone Miner. Metab..

[51] Musumeci G., Loreto C., Carnazza M.L., Coppolino F., Cardile V., Leonardi R. (2011). Lubricin is expressed in chondrocytes derived from osteoarthritic cartilage encapsulated in poly (ethylene glycol) diacrylate scaffold. Eur. J. Histochem..

[52] Musumeci G., Mobasheri A., Trovato F.M., Szychlinska M.A., Graziano A.C.E., Lo Furno D., Avola R., Mangano S., Giuffrida R., Cardile R. (2014). Biosynthesis of collagen I, II, RUNX2 and lubricin at different time points of chondrogenic differentiation in a 3D *in vitro* model of human mesenchymal stem cells derived from adipose tissue. Acta Histochem..

[53] Musumeci G., Lo Furno D., Loreto C., Giuffrida R., Caggia S., Leonardi R., Cardile V. (2011). Mesenchymal stem cells from adipose tissue which have been differentiated into chondrocytes in three-dimensional culture express lubricin. Exp. Biol. Med..

[54] Leonardi R., Rusu M.C., Loreto F., Loreto C., Musumeci G. (2011). Immunolocalization and expression of lubricin in the bilaminar zone of the human temporomandibular joint disc. Acta Histochem..

[55] Leonardi R., Musumeci G., Sicurezza E., Loreto C. (2012). Lubricin in human temporomandibular joint disc: An immunohistochemical study. Arch. Oral Biol..

[56] Leonardi R., Loreto C., Talic N., Caltabiano R., Musumeci G. (2012). Immunolocalization of lubricin in the rat periodontal ligament during experimental tooth movement. Acta Histochem..

[57] Musumeci G., Trovato F.M., Loreto C., Leonardi R., Szychlinska M.A., Castorina S., Mobasheri A. (2014). Lubricin expression in human osteoarthritic knee meniscus and synovial fluid: A morphological, immunohistochemical and biochemical study. Acta Histochem..

[58] Musumeci G., Castrogiovanni P., Trovato F.M., Imbesi R., Giunta S., Szychlinska M.A., Loreto C., Castorina S., Mobasheri A. (2015). Moderate physical activity ameliorates cartilage degeneration in a rat model of aging: A study on lubricin expression. Scand. J. Med. Sci. Sports.

[59] Musumeci G., Trovato F.M., Pichler K., Weinberg A.M., Loreto C., Castrogiovanni P. (2013). Extra-virgin olive oil diet and mild physical activity prevent cartilage degeneration in an osteoarthritis model: An *in vivo* and *in vitro* study on lubricin expression. J. Nutr. Biochem..

[60] Pichler K., Loreto C., Leonardi R., Reuber T., Weinberg A.M., Musumeci G. (2013). In rat with glucocorticoid-induced osteoporosis, RANKL is downregulated in bone cells by physical activity (treadmill and vibration stimulation training). Histol. Histopathol..

[61] Kurz B., Lemke A., Kehn M., Domm C., Patwari P., Frank E.H., Grodzinsky A.J., Schünke M. (2004). Influence of tissue maturation and antioxidants on the apoptotic response of articular cartilage after injurious compression. Arthritis Rheum..

[62] Sui Y., Lee J.H., DiMicco M.A., Vanderploeg E.J., Blake S.M., Hung H.H., Plaas A.H., James I.E., Song X.Y., Lark M.W. (2009). Mechanical injury potentiates proteoglycan catabolism induced by interleukin-6 with soluble interleukin-6 receptor and tumor necrosis factor α in immature bovine and adult human articular cartilage. Arthritis Rheum..

[63] Stevens A.L., Wishnok J.S., Chai D.H., Grodzinsky A.J., Tannenbaum S.R. (2008). A sodium dodecyl sulfate-polyacrylamide gel electrophoresis-liquid chromatography tandem mass spectrometry analysis of bovine cartilage tissue response to mechanical compression injury and the inflammatory cytokines tumor necrosis factor α and interleukin-1β. Arthritis Rheum..

[64] Dang A.C., Kim H.T. (2009). Chondrocyte apoptosis after simulated intraarticular fracture: A comparison of histologic detection methods. Clin. Orthop. Relat. Res..

[65] Kong D., Zheng T., Zhang M., Wang D., Du S., Li X., Fang J., Cao X. (2013). Static mechanical stress induces apoptosis in rat endplate chondrocytes through MAPK and mitochondria-dependent caspase activation signaling pathways. PLoS ONE.

[66] Zaman F., Chrysis D., Huntjens K., Chagin A., Takigawa M., Fadeel B., Sävendahl L. (2014). Dexamethasone differentially regulates Bcl-2 family proteins in human proliferative chondrocytes: Role of pro-apoptotic Bid. Toxicol. Lett..

[67] Echtermeyer F., Bertrand J., Dreier R., Meinecke I., Neugebauer K., Fuerst M., Lee Y.J., Song Y.W., Herzog C., Theilmeier G., Pap T. (2009). Syndecan-4 regulates ADAMTS-5 activation and cartilage breakdown in osteoarthritis. Nat. Med..

[68] Glasson S.S., Askew R., Sheppard B., Carito B., Blanchet T., Ma H.L., Flannery C.R., Peluso D., Kanki K., Yang Z., Majumdar M.K. (2005). Deletion of active ADAMTS5 prevents cartilage degradation in a murine model of osteoarthritis. Nature.

[69] Kon S., Ikesue M., Kimura C., Aoki M., Nakayama Y., Saito Y., Kurotaki D., Diao H., Matsui Y., Segawa T. (2008). Syndecan-4 protects against osteopontin-mediated acute hepatic injury by masking functional domains of osteopontin. J. Exp. Med..

[70] Matsui Y., Iwasaki N., Kon S., Takahashi D., Morimoto J., Matsui Y., Denhardt D.T., Rittling S., Minami A., Uede T. (2009). Accelerated development of aging-associated and instability-induced osteoarthritis in osteopontin-deficient mice. Arthritis Rheum..

[71] Vicencio J.M., Galluzzi L., Tajeddine N., Ortiz C., Criollo A., Tasdemir E., Morselli E., Ben Younes A., Maiuri M.C., Lavandero S. (2008). Senescence, apoptosis or autophagy? When a damaged cell must decide its path—A mini-review. Gerontology.

[72] Almonte-Becerril M., Navarro-Garcia F., Gonzalez-Robles A., Vega-Lopez M.A., Lavalle C., Kouri J.B. (2010). Cell death of chondrocytes is a combination between apoptosis and autophagy during the pathogenesis of Osteoarthritis within an experimental model. Apoptosis.

[73] Musumeci G., Szychlinska M.A., Mobasheri A. (2015). Age-related degeneration of articular cartilage in the pathogenesis of osteoarthritis: Molecular markers of senescent chondrocytes. Histol. Histopathol..

[74] Caramés B., Taniguchi N., Otsuki S., Blanco F.J., Lotz M. (2010). Autophagy is a protective mechanism in normal cartilage, and its aging-related loss is linked with cell death and osteoarthritis. Arthritis Rheum..

[75] Salminen A., Kaarniranta K., Kauppinen A. (2012). Inflammaging: Disturbed interplay between autophagy and inflammasomes. Aging.

[76] Lotz M.K., Caramés B. (2011). Autophagy and cartilage homeostasis mechanisms in joint health, aging and OA. Nat. Rev. Rheumatol..

[77] Li X., Liu F., Liang W., Ye H., Li H., Yu F., Chen J., Chen W., Lin R., Zheng C. (2014). Tougu Xiaotong capsule promotes chondrocyte autophagy by regulating the Atg12/LC3 conjugation systems. Int. J. Mol. Med..

[78] Zhang F.J., Luo W., Lei G.H. (2015). Role of HIF-1α and HIF-2α in osteoarthritis. Jt. Bone Spine.

[79] Takayama K., Kawakami Y., Kobayashi M., Greco N., Cummins J.H., Matsushita T., Kuroda R., Kurosaka M., Fu F.H., Huard J. (2014). Local intra-articular injection of rapamycin delays articular cartilage degeneration in a murine model of osteoarthritis. Arthritis Res. Ther..

[80] Caramés B., Kiosses W.B., Akasaki Y., Brinson D.C., Eap W., Koziol J., Lotz M.K. (2013). Glucosamine activates autophagy *in vitro* and *in vivo*. Arthritis Rheum..

